# Impact of different exercise training modalities on the coronary collateral circulation and plaque composition in patients with significant coronary artery disease (EXCITE trial): study protocol for a randomized controlled trial

**DOI:** 10.1186/1745-6215-13-167

**Published:** 2012-09-14

**Authors:** Madlen Uhlemann, Volker Adams, Karsten Lenk, Axel Linke, Sandra Erbs, Jennifer Adam, Holger Thiele, Thomas Hilberg, Matthias Gutberlet, Martin Grunze, Gerhard C Schuler, Sven Möbius-Winkler

**Affiliations:** 1 Sports medicine, University Wuppertal, Wuppertal, Germany; 2 Department for Radiology, Heart Centre, University of Leipzig, Leipzig, Germany; 3 MediClin-Klinikum Trassenheide, Trassenheide, Germany; 4 Department of Internal Medicine/Cardiology, Heart Centre, University of Leipzig, Strümpellstrasse 39, Leipzig, 04289, Germany

**Keywords:** Atherogenesis, Coronary artery disease, Coronary collateral blood flow, Collateral circulation, Exercise training, Interval training, Pathophysiology

## Abstract

**Background:**

Exercise training (ET) in addition to optimal medical therapy (OMT) in patients with stable coronary artery disease (CAD) has been demonstrated to be superior to percutaneous coronary interventions (PCI) with respect to the composite endpoint of death, myocardial infarction, stroke, revascularization and hospitalization due to worsening of angina. One mechanism leading to this superiority discussed in the literature is the increase in coronary collateral blood flow due to ET. Until now, data demonstrating the positive effect of ET on the collateral blood flow and the functional capacity of the coronary collateral circulation are still lacking.

**Methods/design:**

The EXCITE trial is a three-armed randomized, prospective, single-center, open-label, controlled study enrolling 60 patients with stable CAD and at least one significant coronary stenosis (fractional flow reserve ≤0.75). The study is designed to compare the influence and efficacy of two different 4-week ET programs [high-intensity interval trainings (IT) versus moderate-intensity exercise training (MT) in addition to OMT] versus OMT only on collateral blood flow (CBF). The primary efficacy endpoint is the change of the CBF of the target vessel after 4 weeks as assessed by coronary catheterization with a pressure wire during interruption of the antegrade flow of the target vessel by balloon occlusion. Secondary endpoints include the change in plaque composition as assessed by intravascular ultrasound (IVUS) after 4 weeks, myocardial perfusion as analyzed in MRI after 4 weeks and 12 months, peak oxygen uptake (V02 peak), change in endothelial function and biomarkers after 4 weeks, 3, 6 and 12 months. The safety endpoint addresses major adverse cardiovascular events (death from cardiovascular cause, myocardial infarction, stroke, TIA, target vessel revascularization or hospitalization) after 12 months.

**Discussion:**

The trial investigates whether ET for 4 weeks increases the CBF in patients with significant CAD compared to a sedentary control group. It also examines the impact of two intensities of ET on the CBF as well as the histological plaque composition. The trial started recruitment in June 2009 and will complete recruitment until June 2012. First results are expected in December 2012 (4-week follow-up), final results (12-month long-term secondary endpoint) in December 2013.

**Trial registration:**

Clinical trial registration information-URL: http://www.clinicaltrials.gov.

Unique identifier: NCT01209637

## Background

Coronary artery disease (CAD) still remains one of the leading causes of mortality in many countries with more than 17 million deaths worldwide (WHO 2008). CAD is a result of atherosclerosis, which has been interpreted to be due to endothelial dysfunction and inflammatory reaction [1,2]. Percutaneous coronary interventions (PCI) have been shown to be favorable with respect to major cardiac events in patients with acute coronary syndromes [3,4]. In patients with stable CAD, the benefits of coronary revascularization are less clear. In particular, there is no reported advantage with respect to major cardiovascular events and mortality compared to medical therapy only in patients with stable angina [5-10].

Several randomized trials compared PCI in addition to an optimal medical therapy (OMT) versus OMT only in patients with stable CAD. The COURAGE trial is the largest randomized study comparing an OMT only with a PCI plus OMT in patients with stable CAD. This milestone trial showed comparable efficacy between the two strategies with regard to a combined endpoint of death, myocardial infarction and stroke [11-16].

In contrast, Kastrati and co-workers published a meta-analysis of 17 randomized trials comparing OMT with a PCI-based invasive strategy in 7,513 patients with stable CAD and symptoms or signs of myocardial ischemia [17]. The major finding of this analysis was that PCI may improve patients’ long-term survival compared to OMT with a 20% reduction of the primary endpoint of all-cause death in the PCI group. However, of all the trials included, three also enrolled unstable patients presenting with acute coronary syndrome (DANAMI, TIME, SWISSI II).

Since the first description of the coronary collateral circulation by Blumgart et al. in 1948 [18], extensive investigations have been done to quantify the coronary circulation as well as to assess its anatomical contribution and functional capacity [19]. Berry and co-workers [20] reported the presence of coronary collateral anastomoses to be a prognostically important adaption in patients with CAD.

Seiler et al. [21] demonstrated reduced long-term mortality in patients with stable CAD and well-developed coronary collateral circulation. Moreover, in patients with acute ST elevation myocardial infarction, extensive collateral flow before reperfusion has been shown to be a predictor for a favorable long-term outcome [22].

### Exercise training and CAD

Physical activity has been well documented to reduce the cardiovascular event rate in a long-term perspective [23,24]. In particular, Myers and co-workers reported a strong inverse and graded reduction of mortality in healthy subjects and patients with known CAD with increased physical fitness [25,26].

As previously reported in a randomized trial enrolling 101 male participants, a combination of exercise training (ET) and OMT in patients with stable CAD was associated with a higher event-free survival and an increased maximal oxygen-uptake as compared to PCI + OMT after 1 year of treatment [27]. Several mechanisms are reported to be responsible for the improvement in event-free survival after ET. Besides a reduction of cardiovascular risk factors, an improvement of endothelial function was documented after 4 weeks of ET [28]. An increase of coronary collaterals was often hypothesized, but to date there are no prospective studies available demonstrating a beneficial effect of a specific therapy on the number of pre-existing coronary collaterals or an induction of angiogenesis of collateral vessels in human beings. However, currently no clear consensus exists about how the best physiological adaption can be achieved with different ET modalities regarding the coronary collateral blood flow (CBF) [interval training (IT) versus moderate-intensity continuous exercise training (MT)]. In a recently published review, a strategy of IT alone or in combination with resistance training resulted in significantly greater effects on cardiorespiratory fitness and endothelial function compared to MT [29]. Hambrecht et al. reported a regression of coronary lesions only in patients performing regular physical exercise 5–6 h/week expending an average of 2,200 kcal/week [30].

### Assessment of the collateral blood flow

In the past, there has been growing interest in investigating coronary collateralization [31,32], and several methods have been used to assess the CBF (angiographic guidance versus hemodynamic evaluation).

In 1995, Niebauer and co-workers published a randomized study comparing intensive exercise training of >3 h/week in addition to a low-fat diet with a sedentary control group in regard to the change of the collateral formation [33]. After 1 year, the study failed to show a difference in collateral blood supply between the two groups. This result might have been due to the angiographic evaluation of collateral vessels based only on QCA analysis.

In the 1990s, the method of measuring the fractional flow reserve (FFR) as an invasive pressure-derived index was reported by Pijls and De Bruyne [34,35]. This method allows a measurement of the pressure distal to a coronary stenosis.

Animal studies in healthy dogs [36,37] showed a significant increase in CBF in those who underwent treadmill training (75 min/day, 5 days/week) for 12 weeks. Beside a quantitative increase of collateral vessels, a growth of the diameter of epicardial collaterals was reported.

Compared to angiography-based QCA, measurement of the CBF after an artificial vessel occlusion with a coronary pressure wire seems to be a more functional approach to assess collateral vessels. This method was first described by Pijls et al. [35] as a quantitative assessment of recruitable CBF in human beings. In 120 consecutive patients undergoing elective coronary angiography, an invasive measurement of the mean arterial pressure (P_ao)_, coronary wedge pressure and central venous pressure (CVP) was performed at balloon inflations of 2 min (P_occlusion_). As described by Zbinden et al., the coronary flow index (CFI) is calculated as follows: $\left(P_{\text{occlusion}}-\text{CVP}\right)/\left(P_{\text{ao}}-\text{CVP}\right)$[38]. Zbinden and co-workers have demonstrated an increase of the collateral flow index of normal and stenotic vessels after 3 months of endurance training [35,38]. After PCI of the target vessel, a measurement of the CBF with a pressure wire under vessel occlusion with a balloon was performed. After successful PCI without residual stenosis, patients participated in an exercise program for up to 3 months three times a week. After 3 months, the prevention of existing collateral vessels after PCI with ET was reported. However, some limitations of the study need to be addressed. First, because of its non-randomized study design, a selection bias cannot be ruled out. Second, the initial coronary stenosis leading to angina and cardiac catheterization was treated by PCI before starting the ET program. By treating the stenosis, the ischemic trigger to increase angiogenesis might be reduced. This is partially explained by a reported increase of VEGF levels and circulating progenitor cells and an enhanced integration of EPCs into the endothelial cell layer after performing a 4-week ischemic ET program [39]. The authors did not report in how many patients a bare metal stent or a drug-eluting stent was implanted. This issue might have been important considering the initial worsening of endothelial function of PCI with a drug-eluting stent.

Buschmann et al. recently published a study analyzing the impact of treatment with external counterpulsation on the fractional flow reserve, and they reported a stimulation of coronary angiogenesis in patients with stable CAD after treatment with external counterpulsation for 7 weeks [40]. Again, the non-randomized study design and the lack of a real sham-group are limiting factors of the study.

Meier at al. [41] published a meta-analysis enrolling 6,529 subjects exploring the impact of coronary collaterals on mortality. It has been shown that the presence of high coronary collateralization indicates a 36% reduced mortality risk. The same research group reported a significantly reduced 10-year survival rate in patients with low CBF (CBF <0.25) compared to those with a well-developed collateral circulation (CBF ≥0.25) [42].

Pohl and co-workers [43] investigated the effect of dynamic handgrip exercise on the collateral blood flow in acute coronary occlusions. They found a significant increase of the CBF in patients undergoing PCI performing physical exercise for 3 min compared to a sedentary control group. The CBF was measured at the start and end of two 1-min coronary occlusions.

In addition, Wustmann et al. [44] elucidated the CBF in normal coronary arteries without stenotic lesions. They reported an immediately recruitable CBF that is sufficient to prevent myocardial ischemia during coronary balloon occlusion in 20% of patients without coronary stenosis.

Currently, little is known about the direct effect of physical exercise training on collateral formation, and the prognostic relevance of the CBF is still discussed controversially. Currently, there is no adequately powered clinical trial to assess the effect of different modalities of exercise training on the CBF (high-intensity IT versus MT) in addition to an OMT.

### Hypothesis

Therefore, the *Leipzig EXerCIse Training versus MEdical Management* (EXCITE) trial is designed to test the hypothesis that a 4-week ET therapy in addition to OMT can increase coronary CBF compared to baseline measurements. In a second step analysis, the impact of different ET intensities (IT versus MT) on the change of CBF will be elucidated.

## Methods/design

### Study objectives

The EXCITE study is a three-armed, randomized, prospective, open-label, controlled trial in patients with stable CAD and a significant coronary artery stenosis of at least one major epicardial vessel. The severity of the coronary stenosis is assessed by measurement of the FFR. An FFR of ≤0.75 of at least one coronary stenosis is feasible for study inclusion. The aim of the study is to investigate the efficacy of 4 weeks of intensive exercise training in addition to OMT on the amount of CBF compared to a sedentary control group with OMT only. It will determine if exercise training is superior to standard medical therapy in increasing the CBF. Moreover, the Excite trial wants to demonstrate the superiority of a strategy of intensive interval exercise training compared to moderate-intensity exercise training. The trial is a proof-of-concept study and registered under http://www.clinicaltrial.gov: NCT01209637.

### Primary and secondary outcomes

The primary study endpoint of the EXCITE trial is the change in the coronary CBF index after 4 weeks of therapy among the three treatment groups (Table 1).

**Table 1:** Primary and secondary endpoints

**Primary**	**Change in CBI after 4 weeks of treatment**
Secondary	1. Change in plaque morphology by virtual
	histology at 4 weeks as assessed by IVUS
	2. Change in myocardial perfusion at 4 weeks and
	12 months as assessed by adenosine MRI
	3. MACE at 1, 3, 6 and 12 months
	4. Hospitalization due to cardiovascular causes
	5. Change in atherosclerotic parameters (hs-CRP,
	ADMA, endostatin, EPC, ox LDL, cytokines,
	adiponectine), lipid status and glucose status
	6. Change in exercise capacity (ergospirometry) and
	V0_2_ peak

The secondary endpoints include the change in the tissue composition of the target lesion after 4 weeks of exercise training compared to optimal medical therapy only.

Other secondary endpoints are displayed in Table 1. To confirm a potential advantage by long-term follow-up, a 12-month follow-up will be performed in the course of the secondary endpoint evaluation.

Safety assessment includes bleeding complications related to the invasive measurement of the CBF according to the GUSTO criteria [45], stroke and renal failure.

### Patient population

The study population will consist of 60 patients with stable significant CAD enrolled at the University of Leipzig Heart Centre, Leipzig, Germany.

Patients are eligible if they are >18 and ≤75 years of age and have a significant coronary artery stenosis with a FFR ≤0.75 in at least one coronary artery.

The exclusion criteria reflect known contraindications for exercise training or application of adenosine. Eligible patients have to present with stable angina for at least 8 weeks and need to have ≥1 focal coronary luminal stenosis of more than 50% eligible for PCI. All patients have to be able to perform an ET program with a threshold of at least 50 Watts (W) without angina pectoris or signs of ischemia. Inclusion and exclusion criteria are demonstrated in Tables 2 and 3. Patients have to express willingness to participate in the study for 12 months. Patients presenting with a history of acute coronary syndromes within the past 2 months, significant valve disease, an LV ejection fraction of ≤40% or contraindications to adenosine stress are not eligible. In addition, patients with significant left main coronary disease or an ostial lesion are excluded.

**Table 2:** Inclusion criteria

**Inclusion criteria**
1. Age >18 and <76 years
2. Stable angina pectoris CCS 1-3
3. Luminal stenosis of ≥50% in at least one major epicardial vessel eligible for PCI
4. FFR ≤0.75 in the target vessel
5. Angina threshold ≥50 W
6. Patients have to sign the informed consent form

**Table 3:** Exclusion criteria

**Exclusion criteria**
1. Left main coronary artery stenosis >50%
2.High-grade stenosis (>75%) of the proximal left anterior descending artery
3. Stenosis with a minimum diameter of <2 mm
4. Acute coronary syndromes or recent myocardial infarction within the past 2 months
5. LV-EF ≤40%
6. Significant valvular heart disease
7. Pregnancy
8. Contraindication for application of adenosine:
AV block II and III°
Sick sinus syndrome
Long QT
Asthma bronchiale
9. Limitation of physical activity for reasons not due to coronary artery disease
10. Life expectancy <1 year
11 Substance abuse (drug or alcohol) problem within the previous 6 months

### Study examinations

**Figure 1:**
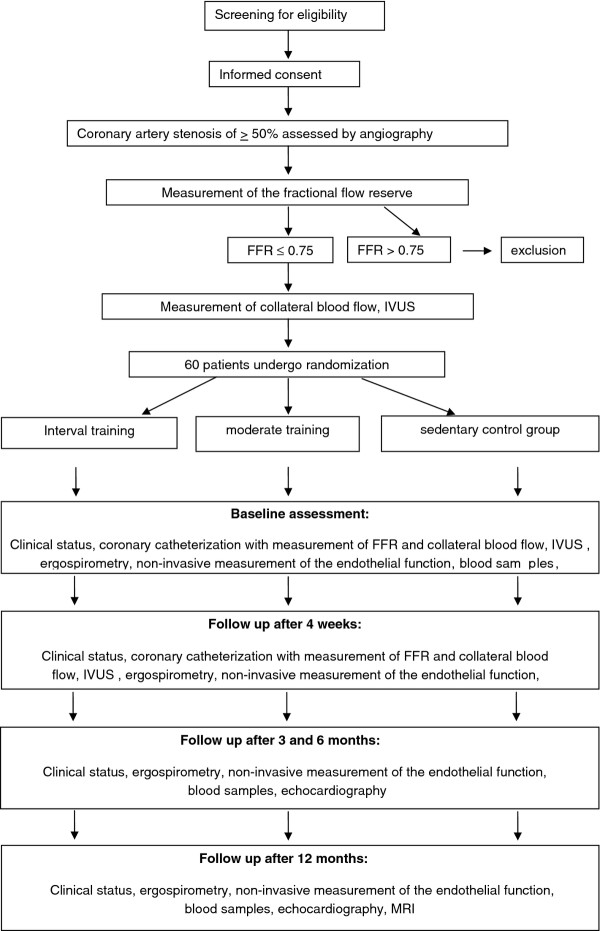
Study flow chart.

After the patient has provided written informed consent, a cardiac catheterization with a clinical indication is performed. In case of a coronary artery stenosis >50%, a measurement of FFR will be performed. Only target vessels that are feasible for PCI with a minimum diameter of ≥2 mm will be included. Therefore, patients with a coronary stenosis of <2 mm will be excluded. Patients with an FFR of ≤0.75 will be enrolled in the study and randomly assigned by a computerized system in a 1:1:1 ratio to one of the three treatment groups (20 patients per group) with stratification according to age and sex. The study flow chart is shown in Figure 1.

### Measurement of FFR

Coronary angiography will be performed with 6-French catheters at baseline and after the 4-week intervention. The FFR is the maximal achievable flow, which determines the functional capacity. The FFR is defined as the mean distal coronary pressure divided by the mean proximal coronary or aortic pressure during maximal hyperemia [46-48]. The measurements will be performed using a coronary pressure guide wire (Prime Wire, Volcano Corp., Rancho Cardova, CA, USA, or Radi Pressure Wire Certus, St. Jude Medical, Sweden). Maximal hyperemia will be achieved by intravenous application of adenosine, administered via a right femoral vein in dose of 140 μg/kg body weight/min.

Measurement of the FFR will be done after application of adenosine infusion for approximately 2 min when a steady state has been reached. Right atrial pressure will be measured via the femoral vein in a standard manner.

### Measurement of the coronary collateral circulation

The primary study endpoint (change in CBF) will be assessed by sensor-tipped guide wires during vessel occlusion with a balloon catheter for 1 min.

Therefore, a standard PCI balloon with a diameter 0.5 mm larger than the diameter of the target vessel is delivered via the FFR guide wire and will be inflated proximal to the coronary lesion within an angiographic healthy coronary segment with a low pressure of 4–6 atm to interrupt antegrade blood flow of the vessel during maximal hyperemia. Contrast dye is injected to show the interruption of the antegrade blood flow. During the measurement, the blood pressure of the target vessel distal to the coronary stenosis is continuously monitored as well as the systemic blood pressure.

To differentiate if the results after 4 weeks of therapy are due to CAD or physiological adaptations, the same measurements will be performed in a coronary vessel free of coronary stenosis (the reference vessel).

After 4 weeks of treatment, measurements will be repeated in the same coronary segments.

Cardiac catheterization as well as measurement of the CBF will be performed by three very experienced invasive cardiologists with expertise in hemodynamic analysis. Nevertheless, the procedure may carry some risks such as dissections of the coronary artery by the pressure wire, development of the thrombus burden, stroke or myocardial infarction during vessel occlusion. Application of adenosine intravenously can result in a complete atrioventricular block with short asystole.

### Assessment of clinical symptoms and patient treatment

All patients undergo detailed assessment of medical history as well as physical examination at baseline and after 1, 3, 6 and 12 months. In patients without known diabetes, an oral glucose tolerance test will be performed before inclusion.

At baseline, 1, 3, 6 and 12 months, the angina pectoris status will be documented according to CCS class 1–4 by a physician blinded to the treatment group. In addition, a symptom-limited bicycle ergospirometry and an echocardiography will be conducted.

An OMT is adjusted according to current clinical practice guidelines in each group at least 2 weeks before initial measurements were obtained. Statins, ß blockers, angiotensin-converting enzyme inhibitors or angiotensin-1 antagonists are obligatory. In addition, patients are treated with an antiplatelet therapy with aspirin 100 mg daily or, if the patient has undergone a previous PCI, a dual antiplatelet therapy will be administered according to current guidelines.

All patients enrolled in the study will be followed for 12 months. At any time during the study period, each patient can withdraw the informed consent. A patient with signs of clinically worsening symptoms will be thoroughly evaluated by the investigator, and if clinically indicated a PCI will be performed. In patients presenting with an acute coronary syndrome, a coronary angiography will be performed immediately, and the target lesion will be treated by PCI. The study will be discontinued for a given patient if continuing would result in a significant safety risk.

### Qualitative Comparative Analysis (QCA Analysis)

QCA measurements of the target lesion and the healthy reference coronary artery will be obtained at baseline and after 4 weeks respectively by two independent operators using the Medis Software (Medis Medical Imaging Systems, QAngio XA, version 7.2).

The following parameters will be measured: lesion lengths (mm), minimum/maximum lumen diameter (mm), target stenosis (%) and the diameter of the reference coronary artery at the location of FFR measurement (mm).

### Intravascular ultrasound examination (IVUS sub-study)

IVUS imaging with an analysis of the virtual histology of the coronary plaque has the potential to overt possible effects of the specific therapy of the study with respect to change in the plaque composition and plaque volume of the target lesion.

Therefore, an IVUS with virtual histology analysis will be performed at baseline and after 4 weeks of therapy. After measurement of the FFR and CBI, an Eagle Eye^R^ Gold Intravascular Ultrasound Imaging Catheter (Volcano Corporation, Rancho Cardova, CA, USA) will be inserted distal to the target lesion at a landmark side branch and pulled back via a motorized transducer pullback device for a length of at least 4 cm. ECG-triggered virtual histology is performed to analyze the tissue composition.

The details on IVUS image acquisition and analysis have been reported previously [49-53]. The percentage of atheroma volume, total plaque burden, minimal lumen area (mm^2^), lesion lengths (mm) and differentiation of plaque tissue (%) will be measured [54].

### Adenosine MR perfusion imaging (MRI sub-study)

A cine MRI and an adenosine perfusion MR will be performed at baseline, 4 weeks and 12 months. The aim of this sub-study is to investigate whether MR perfusion imaging can predict myocardial perfusion defects, collateral circulation, LV function and volumes after 4 weeks of exercise training compared to a sedentary control group.

Imaging will be performed with a 1.5-T scanner (Philips Intera CV, Best, The Netherlands) in a standard fashion. After 4 min of adenosine infusion, a perfusion sequence will be started according to a standard protocol. All measurements will be performed by blinded operators with excellent intra- and interobserver variabilities and excellent reproducibility. Exclusion criteria for the MRI sub-study are (1) severe claustrophobia, (2) implantable pacemaker and defibrillator, and (3) contraindications for adenosine (first and second degree AV block, sick sinus syndrome, long QT syndrome, asthma bronchiale).

### Exercise testing

All patients will perform a cardiopulmonary exercise test on a bicycle ergospirometer (Ergoline, Bitz, Germany) to determine the individual exercise capacity and the peak oxygen uptake (V0_2_ peak), angina threshold and ischemic threshold. The step protocol starts at 25 W and increases by steps of 25 W every 3 min. The Zan SpiroErgo 600 (Zan, Winkling, Austria) is used to measure breath-by-breath gas exchange levels of ventilation (E), oxygen uptake and carbon dioxide production (CO_2_). Important measured parameters are the exercise capacity (Watts), angina pectoris threshold, ischemic threshold, V0_2_ max/peak, time to discontinuation (T max) and time to anaerobic threshold (T RQ1). After 4 weeks, 3, 6 and 12 months, the test will be repeated.

### Exercise training program

Patients randomized in the IT group perform exercise training under supervision by experienced physicians and exercise physiologists four times daily for 30 min, five times a week for a total of 4 weeks. The training program is intended to be a high-intensity training with partial IT up to 95% of the individual ischemia-/angina-free exercise capacity.

Patients randomized in the MT group will undergo a multimodal lifestyle intervention in a specialized hospitalized rehabilitation unit. These patients will exercise 6–8 sessions per day (20 min each) under close medical supervision at a maximum level of 70% of their individual angina-/ischemia-free exercise threshold. Additionally, they will be in lifestyle modification as well as individual physiotherapeutic therapies.

Patients assigned to the sedentary control group receive an OMT and are encouraged to carry out regular ET according to current guidelines (2–3 sessions per week, 20–30 min each). They are supervised by their general practitioner.

### Laboratory values and biomarkers

Blood samples will be taken and analyzed at baseline, at 1, 3, 6 and 12 months. Routine measurements of lipid and glucose metabolism will be performed. In addition, the amount of circulating endothelial progenitor cells (EPCs) will be quantified by flow cytometry as recently described [55,56].

Asymmetric dimethylarginine (ADMA) and endostatin will be measured using a commercial available ELISA kit (for ADMA: Immundiagnostik AG, Bensheim, Germany; for Endostatin: Antigenix American Incorporation) following the manufacturer’s instructions.

The association between interleukin-6 and tumor necrosis factor-alpha to the CBF will be determined.

### Long-term follow-up

Follow-up will be conducted after 3, 6 and 12 months as ambulatory visits at our institution. In case of any events, these will be verified by hospital charts or direct contact with the treating physician.

### Data and statistical analysis

In the absence of randomized trials and lacking data regarding ET and its impact on the collateral blood supply, a power calculation was not possible. Therefore, the Excite trial is designed to be a proof-of-concept study.

Categorical variables are presented as frequencies and percentages, continuous variables as means and standard deviations (SD), or medians and interquartile ranges for variables with skewed distributions. The primary endpoint will be analyzed according to the intention-to-treat principle using the Kruskal-Wallis test. In addition, event rates in all of these three treatment arms will be estimated (95% CI), and the time-to-event rates will be described using the Kaplan-Meier method. The secondary endpoints (Table 3) will be analyzed by appropriate methods according to data type to compare the three treatment arms. If statistically possible, pre-defined subgroup analyses will be performed including patients’ gender, presence of diabetes, amount of pre-existing CBF and FFR of the target lesion at baseline.

## Discussion

The EXCITE trial protocol is focused on patients undergoing cardiac catheterization with stable CAD and hemodynamically significant coronary artery stenosis. According to previous data, there is still uncertainty whether ET increases the coronary collateral circulation. One critical point of previous studies is the angiographic evaluation of collateral vessels only. Therefore, the invasive measurement of the FFR-based CBF is a functional approach with higher accuracy to assess collateral vessels. This might help to differentiate even small effects of the several therapies. So far, FFR-based evaluation of CBF has only been reported in non-randomized trials.

The EXCITE trial is designed as an all-comers study without specific subgroup exclusion in order to receive generalizable results. For more detailed insight, we pre-defined several subgroups.

Nevertheless, within this study two different training programs can be compared with a sedentary control group. This might help to find the most effective training program in patients with stable angina pectoris and significant coronary stenosis. Because of the lack of previous data investigating the CBF, the present study was designed as a proof-of-concept-trial. Therefore, a valid power calculation was not possible.

Blinding to avoid bias might have been beneficial. However, we implemented methods to reduce bias, such as the use of a central computerized randomization system and a blinded MRI and IVUS core laboratory.

To the best of our knowledge, this is the first randomized study comparing the CBF in patients after high- and moderate-intensity 4-week physical exercise training programs compared to OMT only.

## Conclusion

Exercise training results in an increased delivery of blood to myocardial regions in need, but the pathophysiology is still unclear.

The Excite trial was designed to investigate whether intensive exercise training for 4 weeks as IT and MT in comparison to a sedentary control group with OMT only will increase the coronary collateral circulation in patients with stable CAD and significant epicardial stenosis (FFR ≤ 0.75).

## Trial status

Recruitment has commenced in June 2009. We expect to complete recruitment by June 2012. Final results (12-months clinical endpoint) are planned to be published in July 2013.

## Abbreviations

ADMA: Asymmetric dimethylarginine; CAD: Coronary artery disease; CBF: Coronary collateral blood flow; CI: Confidence interval; EPC: Endothelial progenitor cells; ET: Exercise training; FFR: Fractional flow reserve; IT: High-intensity interval trainings; IVUS: Intravascular ultrasound; MACE: Major adverse cardiovascular events; MRI: Magnet resonance imaging; MT: Moderate-intensity exercise training; OMT: Optimal medical therapy; PCI: Percutaneous coronary intervention; QCA: Qualitative comparative analysis; VEGF: Vascular endothelial growth factor.

## Competing interests

None of the authors have competing interests in the context of the EXCITE trial.

## Authors’ contributions

The EXCITE trial Steering Committee is chaired by SM-W and co-chaired by MU, University of Leipzig Heart Centre, Leipzig, Germany. The authors are responsible for designing and conducting the study, statistical analyses, drafting and editing of the article. The study protocol was approved by the ethics committee of the University Leipzig, Germany. There are no sources of funding. All authors read and approved the final manuscript.
